# The Relationship between Seropositive Rheumatoid Arthritis and Congestive Heart Failure: A Nationwide Longitudinal Cohort Study in Korea

**DOI:** 10.3390/jpm14060615

**Published:** 2024-06-08

**Authors:** Yeo Song Kim, Je Beom Hong, Hakyung Kim, Seung Hun Sheen, In-bo Han, Jeong Gyun Kim, Sin Soo Jeun, Seil Sohn

**Affiliations:** 1 Department of Neurosurgery, Incheon St. Mary’s Hospital, College of Medicine, The Catholic University of Korea, Seoul 06591, Republic of Korea; 2 Kangbuk Samsung Hospital, Sungkyunkwan University College of Medicine, Seoul 03181, Republic of Korea; 3 Genome & Health Big Data Branch, Department of Public Health, Graduate School of Public Health, Seoul National University, Seoul 03080, Republic of Korea; 4 Department of Neurosurgery, CHA Bundang Medical Center, CHA University, Seongnam 13496, Republic of Korea; nssheen@cha.ac.kr (S.H.S.);; 5 Department of Neurosurgery, Cheongju St. Mary’s Hospital, Cheongju-si 17319, Republic of Korea; 6 Department of Neurosurgery, Seoul St. Mary’s Hospital, College of Medicine, The Catholic University of Korea, Seoul 06591, Republic of Korea

**Keywords:** seropositive rheumatoid arthritis, congestive heart failure, population, epidemiology

## Abstract

**Objectives:** The aim of this nationwide longitudinal cohort study is to determine the risk of congestive heart failure (CHF) associated with a seropositive rheumatoid arthritis (RA) population in Korea. **Methods:** In this study, National Health Insurance Service-Health Screening Cohort (NHIS-HEALS) data from 2002 to 2003 were used. The cohort was followed up with for 12 years until December of 2015. Seropositive RA was defined as a patient prescribed with a disease-modifying anti-rheumatic drug (DMARD) among patients with the International Classification of Diseases code M05 (seropositive RA). Patients who were diagnosed before 2004 were excluded. The seropositive RA group consisted of 2765 patients, and a total of 13,825 patients were in the control group. The Kaplan–Meier method was used to calculate the 12-year CHF incidence rate for each group. A Cox proportional hazards regression analysis was used to estimate the hazard ratio of CHF. **Results:** The hazard ratio of CHF in the seropositive RA group was 2.41 (95% confidence interval (CI): 1.40–4.14) after adjusting for age and sex. The adjusted hazard ratio of CHF in the seropositive RA group was 2.50 (95% CI: 1.45–4.30) after adjusting for age, sex, income, and comorbidities. In females aged ≥65 and aged <65, the incidence rates in the non-hypertension, non-diabetes mellitus, and non-dyslipidemia subgroups were significantly higher in the seropositive RA group than in the control group. **Conclusions:** This nationwide longitudinal cohort study shows an increased risk of CHF in patients with seropositive RA.

## 1. Introduction

Rheumatoid arthritis (RA) is a chronic systemic inflammatory autoimmune disease that affects the synovial joints, leading to persistent inflammation, pain, and eventual joint destruction, and it is widely believed to have an increased risk of mortality [1,2,3]. The estimated global prevalence of RA is between 0.5% and 2.0%, with both the incidence and prevalence of RA being two to three times higher in women [4]. The estimated prevalence in Korea ranged from 0.27% to 1.85%, which is similar to the global prevalence [4]. Cardiovascular disease has been identified as an important cause of mortality in patients with RA, accounting for 40% of RA mortality, followed closely by cerebrovascular disease [5,6,7,8]. RA has been shown to be an independent risk factor for cardiovascular disease, alongside other well-established risk factors such as aging, hypertension, diabetes, dyslipidemia, obesity, and smoking [9]. Among cardiovascular diseases, congestive heart failure (CHF) is also a fatal disease with a prevalence of approximately 26 million worldwide [10]. In the past, only ischemic heart disease among cardiovascular diseases was associated with RA, while its association with CHF was unclear [9]. However, recent studies have increasingly reported that CHF occurs significantly more frequently in patients with RA. Furthermore, CHF appears to have a greater impact on excessive mortality than ischemic disease in patients with RA [11]. Recent studies have reported that patients with RA with CHF have significantly higher mortality rates compared to those without CHF [12].

Several studies have reported the incidence of CHF in patients with RA. One study conducted a retrospective, population-based, incidence cohort study which included 575 patients with RA and 583 control patients. However, this study was limited by its small sample size, and its focus was on a specific population in Rochester, Minnesota [13]. Other studies used nationwide cohorts in Denmark, USA, and Sweden, but these studies were conducted in the West [14,15,16]. There has been no large-scale study of CHF with RA in Korea. The Korean government provides financial support specifically for the group of patients with seropositive RA among those with RA. Therefore, the diagnosis of patients with seropositive RA is managed very accurately. Therefore, this study aimed to investigate the risk of the incidence of CHF through a nationwide longitudinal cohort study of patients with seropositive RA in Korea.

## 2. Materials and Methods

### 2.1. Data Sources

The Republic of Korea has a mandatory medical insurance system managed by the National Health Insurance Services (NHIS), which ensures that all citizens are registered under this system [17,18,19]. As a part of its services, the NHIS also conducts national health examinations for those over the age of 40. These examinations are conducted every two years for office workers and once a year for non-office workers. To facilitate research and improve public health outcomes, the NHIS established the National Health Insurance Service-Health Screening Cohort (NHIS-HEALS) database, which represents a cohort of individuals who participate in national health examinations [20].

The NHIS-HEALS database classifies diseases according to the International Classification of Diseases (ICD-10) and has extensive information. Researchers who are approved by the official review committee can use this NHIS-HEALS database, and we acquired the rights to use it from the institutional review board of the CHA Bundang Medical Center of CHA University (IRB No. 2020-01-011).

### 2.2. Patient Population

**Figure 1:**
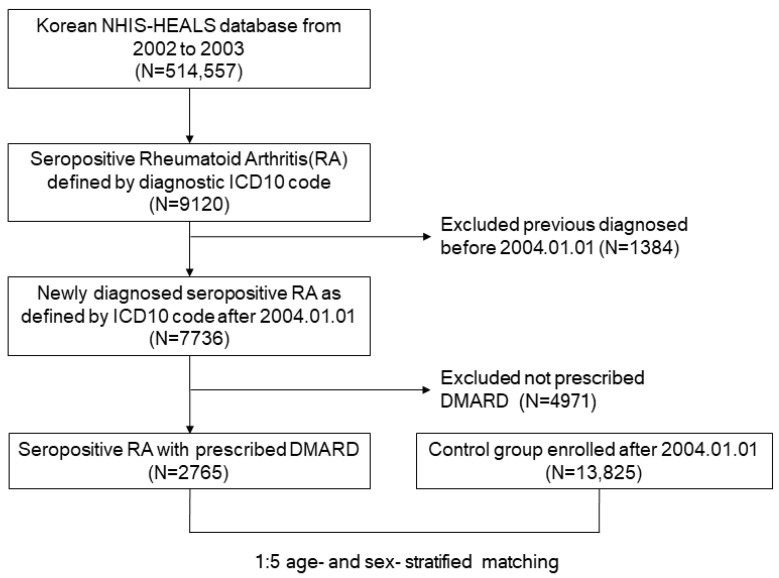
A flowchart of the establishment of this nationwide study. Among the patients with seropositive RA (RA) (*n* = 9120), patients with a previous diagnosis before 2004 were excluded. Subsequently, patients who have not been prescribed with DMARD were excluded.

The NHIS-HEALS cohort included 514,557 participants, approximately 10% of people who participated in health examinations from 2002 to 2003. The cohort consists of individuals aged between 40 and 79 years old, and they were followed up withfor 12 years until December of 2015 [20]. Within this cohort, diseases are classified using ICD-10 codes. The ICD-10 codes of RA were M06 and M05. Specifically, cases of RA where the rheumatoid factor or anti-CCP antibody is positive are classified as seropositive RA. In Korea, patients with a seropositive RA code (ICD-10 code M05) receive financial support from the government. Therefore, doctors are encouraged to make accurate diagnoses concerning patients with seropositive RA, thereby enhancing the reliability of diagnosis data for research purposes. Thus, this study focused on patients with seropositive RA. Initially, 9120 subjects with seropositive RA who were assigned the diagnosis code M05 were extracted from 1 January 2002 to 31 December 2015. Afterwards, 1384 patients who were diagnosed before the start of the study in 2004 were excluded, and only newly confirmed patients with seropositive RA codes were included. A previous paper found that patients with seropositive RA codes and disease-modifying anti-rheumatic drug (DMARD) prescriptions had high sensitivity, a positive predictive value, and a high accuracy of RA diagnosis in Korea [21]. Therefore, after the exclusion of those without a prescription for any DMARD, 2765 subjects with seropositive RA remained (Figure 1). In the next step, age- and sex-stratified matching in a ratio of 1:5 was performed using a greedy digit match algorithm of the R package ‘Match IT’, and 13,825 individuals were chosen as a control group [22,23]. During this process, 2765 patients with seropositive RA were matched with 13,825 controls (Figure 1). Patients in this study were followed up with from the first onset of CHF until death or until the end of the follow-up. The risk of CHF was evaluated after adjusting for age, sex, and comorbidities, including diabetes mellitus, hypertension, and dyslipidemia. 

### 2.3. Definitions of CHF and Comorbidities

A CHF diagnosis was defined as an ICD-10 code of I50 and hospitalization ≥1 day [24,25,26]. During comorbidities, diabetes mellitus was defined as E11–E14; hypertension was defined as I10–I13 and I15; and dyslipidemia was defined as E78 [25,27,28] (Table 1). 

### 2.4. Statistical Analysis

The variables about medical and socioeconomic status and demographic information used in this study are categorical or continuous. Age is a continuous variable, and except for age, all of the variables including sex, age ≥65, income status, diabetes mellitus, hypertension, and dyslipidemia are all categorical. Therefore, to compare the characteristics between the seropositive RA group and the control group, the statistical difference was tested using a two-sample t-test for continuous variables and using Chi-square statistics for categorical variables. The statistical power of the t-test and Chi-square statistics in our study was 1.0. The Kaplan–Meier test was used to estimate the cumulative incidence probabilities of CHF in the seropositive RA and control groups. Multivariate analyses were performed using the Cox proportional hazards regression model to estimate the effects of seropositive RA on CHF. The incidence rate was calculated as the number of cases per 1000 person years. Two Cox proportional hazards regression models were applied to estimate the hazard ratio (HR) and the corresponding 95% confidence intervals (CI). In model 1, age and sex were adjusted. In model 2, age, sex, income, and comorbidities were adjusted. We constructed subgroups of RA and control groups according to sex, age, income, and comorbidities. Age was divided into those over 65 and those under 65 years old. Income was divided into upper/middle and lowest categories. Comorbidities included hypertension, diabetes mellitus, and dyslipidemia. The analyses were performed using R software (version 3.3.3).

## 3. Results

### 3.1. Baseline Characteristics of Subjects

Table 2 shows the baseline characteristics at the time of diagnosis for 2765 patients with seropositive RA and for 13,825 patients in the control group with matching age and sex. Females (73.4%) outnumbered males (26.6%). The mean age of the subjects was 53.5 ± 8.7 years. There were significant differences in terms of the prevalence rates of comorbidities between the seropositive RA and control groups. The group with seropositive RA had a higher rate of hypertension (*p* < 0.0001) than the control group, and the control group had higher rates of diabetes mellitus (*p* < 0.0001) and dyslipidemia (*p* = 0.004) than the seropositive RA group (Table 2).

### 3.2. CHF in the Seropositive RA and Control Groups

**Figure 2:**
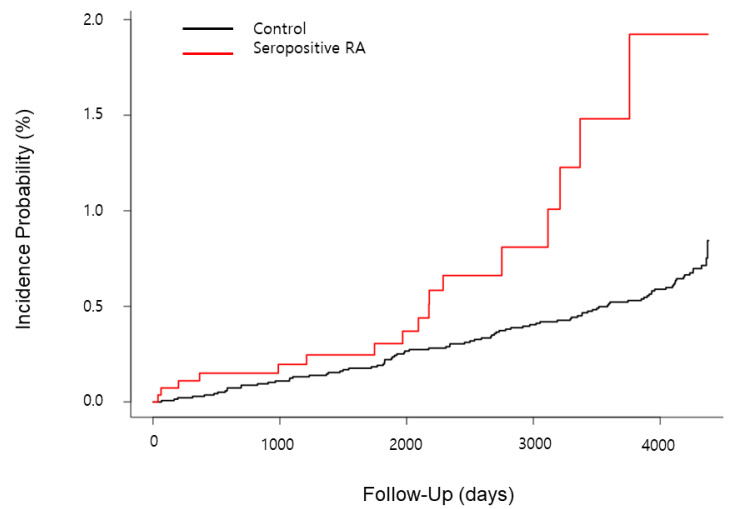
A comparison of the incidence probabilities of CHF in the seropositive RA and control groups. The Kaplan–Meier curves with cumulative hazards of CHF were compared between the seropositive RA and control groups.

The Kaplan–Meier curves show that the seropositive RA group had a higher risk of CHF than the control group (Figure 2). In model 1, the HR of CHF in the group with RA was 2.41 compared to the control (95% CI, 1.40–4.14, Table 3). In model 2, the HR of CHF in the group with RA was 2.50 compared to the control group (95% CI, 1.45–4.30, Table 3). 

### 3.3. Subgroup Analysis of CHF Incidence Rate

The incidence rate of CHF was significantly different between the seropositive RA and control groups among the female subgroup (HR 2.39, 95% CI, 1.29–4.45, Table 4), both age (<65 and ≥65) subgroups (HR 2.60, 95% CI, 1.23–5.50 and HR 2.25, 1.03–4.93, respectively, Table 4), the non-diabetes mellitus subgroup (HR 2.68, 95% CI, 1.54–4.64, Table 4), non-hypertension subgroup (HR 2.99, 95% CI, 1.44–6.22, Table 4), and the non-dyslipidemia subgroup (HR 2.71, 95% CI, 1.54–4.77, Table 4).

## 4. Discussion

We conducted a nationwide longitudinal cohort study based on the NHIS-HEALS database. This study covered all age groups from 40 to 79 years old who were enrolled between 2002 and 2003 and analyzed data between 2004 and 2015. In this nationwide cohort study, the CHF risk is significantly higher in 2765 patients with seropositive RA. Notably, the risk of CHF in patients with seropositive RA is also significantly higher after adjusting for ischemic heart disease risk factors, such as diabetes mellitus, hypertension, and dyslipidemia. 

Several studies reported that the risk of CHF is significantly higher than the control [11,13,14,15,16]. According to Paulo et al., 2005 [13] when comparing 575 patients with RA with 583 controls, the incidence of CHF was twice as high in the patients with RA, and the risk was higher in seropositive patients than in seronegative ones. They also stated that the risk of developing CHF starts immediately after the onset of RA and continues consistently throughout the disease course [13]. Furthermore, they suggested that the increased risk of CHF in patients with RA is due to RA itself rather than cardiovascular risk factors, such as diabetes, smoking, hypertension, or clinical ischemic heart disease [13]. A nationwide study in Denmark compared 24,343 patients with RA with 4,280,882 controls and found that CHF occurred more than twice as often in patients with RA compared to controls [14]. In the United States, a study was conducted on 3,831,006 patients hospitalized with RA, of which 75% were female and 75% were Caucasian. It was also confirmed that CHF, acute myocardial infarction, and atrial fibrillation were significantly increased in this group of patients with RA [15]. In Sweden, non-ischemic CHF and ischemic CHF were investigated separately, with non-ischemic CHF rapidly increasing after the onset of RA, potentially mediated by high inflammatory activity. Ischemic CHF, on the other hand, appeared to occur more slowly over time. Thus, they suggested that patients with RA may develop CHF due to reasons other than ischemic causes [16]. Similar to previous studies, our study also found that the incidence rate of CHF was significantly increased in patients with seropositive RA when adjusting for cardiovascular risk factors. 

We performed a subgroup analysis. And the incidence rate of CHF was significantly higher in the seropositive RA group than the control group among the female subgroup. This result was also consistent with a previously reported study. Khalid et al. reported a meta-analysis study showing a three-fold increased risk of CHF in women with RA [9]. Traditionally, the American College of Cardiology/American Heart Association has considered gender, age, race, cholesterol levels, systolic blood pressure, diabetes, and smoking as risk factors for CHF. And women are assessed to have a higher risk of CHF. The higher incidence of CHF in women with RA might be attributed to these factors. Also, Khalid reported that the risk of CHF gradually increases with age and that there is a higher incidence of CHF in patients with RA compared to controls even in the presence of hypertension or diabetes mellitus [9]. In our study, there was a significant difference in the incidence of CHF among both older and younger patients. However, we did not examine whether CHF increases with age. Additionally, it was found that in patients without diabetes, hypertension, or dyslipidemia, patients with RA had a significantly higher incidence of CHF compared to the control group. However, no statistical difference was observed in patients with diabetes, hypertension, or dyslipidemia. This is likely due to the small number of patients with both diabetes and RA, both hypertension and RA, or both dyslipidemia and RA, indicating a need for further research with a larger dataset in the future. On the other hand, in a cohort study of 2045 patients with RA in the United States, it was found that patients with RA and CHF had significantly higher all-cause mortality (HR 1.60, 95% CI 1.27–2.01) and cardiovascular disease mortality (HR 1.45,95% CI 1.45–3.06). This increased risk is particularly significant among females and individuals aged 65 and older [12]. Therefore, it is necessary to pay more attention to the treatment of patients with RA and CHF, especially in females or older patients. The reason for increased CHF risk in patients with RA is unclear. Previous studies have described several possible mechanisms associated with the increased risk of CHF in patients with RA. Several studies have demonstrated a correlation between RA and CHF as accelerated atherosclerosis due to systemic inflammation [15,29,30]. One of these mechanisms involves the expansion of the CD4+CD28- T cell subset, which is not found in healthy individuals, being significantly expanded in the blood of patients with RA [31]. Furthermore, the expansion of clonal T cells is known to increase the prevalence of atherosclerosis [32]. This resulting atherosclerosis can contribute to the pathogenesis of cardiovascular disease, including CHF [31]. Another study explaining the mechanism of atherosclerosis induction by another inflammatory response indeed showed increased serum cytokines levels in patients with CHF [33]. In particular, it has been reported that cytokines such as tumor necrosis factor- α (TNF- α) contribute to myocardial contraction and cause CHF [16]. Recent studies have suggested that higher levels of inflammation markers in the elderly population may predict the risk of CHF [34]. Additionally, seropositive RA may have the human leukocyte antigen class II histocompatibility, the D-related beta chain (HLA-DRB1) shared epitope gene compared to seronegative RA, and can exhibit a higher cardiovascular mortality rate [35]. On the other hand, RA drugs such as non-steroidal anti-inflammatory drugs (NSAIDs) [36,37], chloroquine, corticosteroids [38,39], D-penicillamine [40], or biologics (TNF-inhibitors) [14] may have cardiotoxic effects and cause an increased risk of CHF [11,13]. However, recent studies suggest that the well-known increased cardiovascular disease risk associated with NSAIDs may not apply to patients with chronic inflammation, including RA [41], and large-scale randomized controlled trials did not report a significant increase in cardiovascular disease events in the RA group treated with celecoxib, naproxen, or ibuprofen [42]. Unfortunately, we were unable to obtain information on the dosages of individual NSAIDs and other drugs. To understand the association between RA and CHF, further research is needed, including research on the types of drug use and the blood levels of inflammation and cholesterol. 

Several limitations of this study should be noted. First, our RA group was limited to seropositive RA, potentially limiting the generalization of the results. Second, NHIS-HEALS only covers those who are 40 to 79 years old, which is a range slightly younger than the general population of Korea [20]. Third, variables in the healthcare claim data cannot exactly reflect patients’ medical statuses, including the medications taken and blood levels [20]. Despite this limitation in mind, this is the largest nationwide longitudinal cohort study to show a correlation between CHF and seropositive RA in patients.

## 5. Conclusions

This nationwide longitudinal cohort study shows an increased risk of CHF in patients with seropositive RA. Clinicians should consider a high risk of CHF, especially in female, non-hypertension, non-diabetes mellitus, and non-dyslipidemia subgroups.

## Figures and Tables

**Table 1:** List of definitions of outcomes and comorbidities.

	Disease (ICD-10 Code)	Additional Conditions
**Past medical history**		
Hypertension	I10-I13, I15	Claims for antihypertensive agents or SBP ≥ 140 mmHg or DBP ≥ 90 mmHg
Type 2 DM	E11–E14	Claims for oral antidiabetic agents or insulin or fasting glucose ≥ 126
Dyslipidemia	E78	Claims for agents for dyslipidemia or total cholesterol ≥ 240
**Socioeconomic status**		
Low income		Composite of lowest quartile of yearly income in addition to medicare beneficiaries
**Outcomes**		
Congestive heart failure	I50	Hospitalization ≥ 1 day

All variables except hypertension and diabetes mellitus were defined when patients had one or more diagnoses during hospitalization or at outpatient clinic. Hypertension and diabetes mellitus were identified to have diseases when patients had either one diagnosis during hospitalization or twice or more diagnoses at outpatient clinics for preventing overestimation of diagnosis. Diabetes was defined as ICD-10 code and above and prescription of anti-diabetic drugs (sulfonylureas, metformin, meglitinides, thiazolidinediones, dipeptidyl peptidase-4 inhibitors, α-glucosidase inhibitors, and insulin). Abbreviations: ICD-10, International Classification of Disease, 10th edition; DM, diabetes mellitus.

**Table 2:** Characteristics of seropositive RA and control group.

Variable	Seropositive RA (n = 2765)	Control (n = 13,825)	*p*-Value
Male, n (%)	735 (26.6)	3675 (26.6)	1
Age, n	53.5 ± 8.7	53.5 ± 8.7	1
AGE ≥ 65, n (%)	352 (12.73)	1760 (12.73)	1
Low Income, n (%)	703 (25.42)	3713 (26.86)	0.126
Diabetes Mellitus, n (%)	173 (6.26)	1396 (10.10)	**<0.0001**
Hypertension, n (%)	833 (30.13)	5038 (26.44)	**<0.0001**
Dyslipidemia, n (%)	430 (15.55)	2466 (17.84)	**0.004**

Bold text indicates statistical significance. Abbreviations: RA, rheumatoid arthritis; CHF, congestive heart failure.

**Table 3:** Adjusted hazard ratio for CHF in seropositive RA and control groups.

Group	No.	Event	Duration (Days)	IR (1000 Person Years)	HR (95% CI)	
					MODEL 1	MODEL 2
**Control**	**13,825**	**91**	**56,009,292**	0.593	1	1
RA	2765	17	5,629,860	1.102	2.41 (1.40, 4.14)	2.50 (1.45, 4.30)

Model 1 was adjusted for age and sex. Model 2 was adjusted for age, sex, income, diabetes mellitus, hypertension, and dyslipidemia. Abbreviations: RA, rheumatoid arthritis; IR, incidence rate; CI, confidence interval; HR, hazard ratio.

**Table 4:** Subgroup analysis of seropositive RA and control groups.

Variables			Seropositive RA		Control	HR (95% CI)
		N	IR (1000 Person Years)	N	IR (1000 Person Years)	
Sex	Male	4	1.057	23	0.591	2.49 (0.82, 7.55)
	Female	13	1.117	68	0.594	2.39 (1.29, 4.44)
Age	<65	9	0.664	45	0.332	2.60 (1.23, 5.50)
	≥65	8	4.313	46	2.552	2.25 (1.03, 4.93)
Diabetes Mellitus	No	17	1.178	78	0.564	2.68 (1.54, 4.64)
	Yes	0	-	13	0.862	-
Hypertension	No	10	0.932	38	0.388	2.99 (1.44, 6.22)
	Yes	7	1.492	53	0.955	2.08 (0.92, 4.71)
Dyslipidemia	No	16	1.234	74	0.588	2.71 (1.54, 4.77)
	Yes	1	0.408	17	0.615	0.89 (0.11, 6.97)

Abbreviations: RA, rheumatoid arthritis; CI, confidence interval; IR, incidence rate; HR, hazard ratio.

## Data Availability

The NHIS-HEALS data were obtained from the NHIS research DB, and due to the lack of patient privacy protection and prior consent for data sharing, the data cannot be shared publicly.
